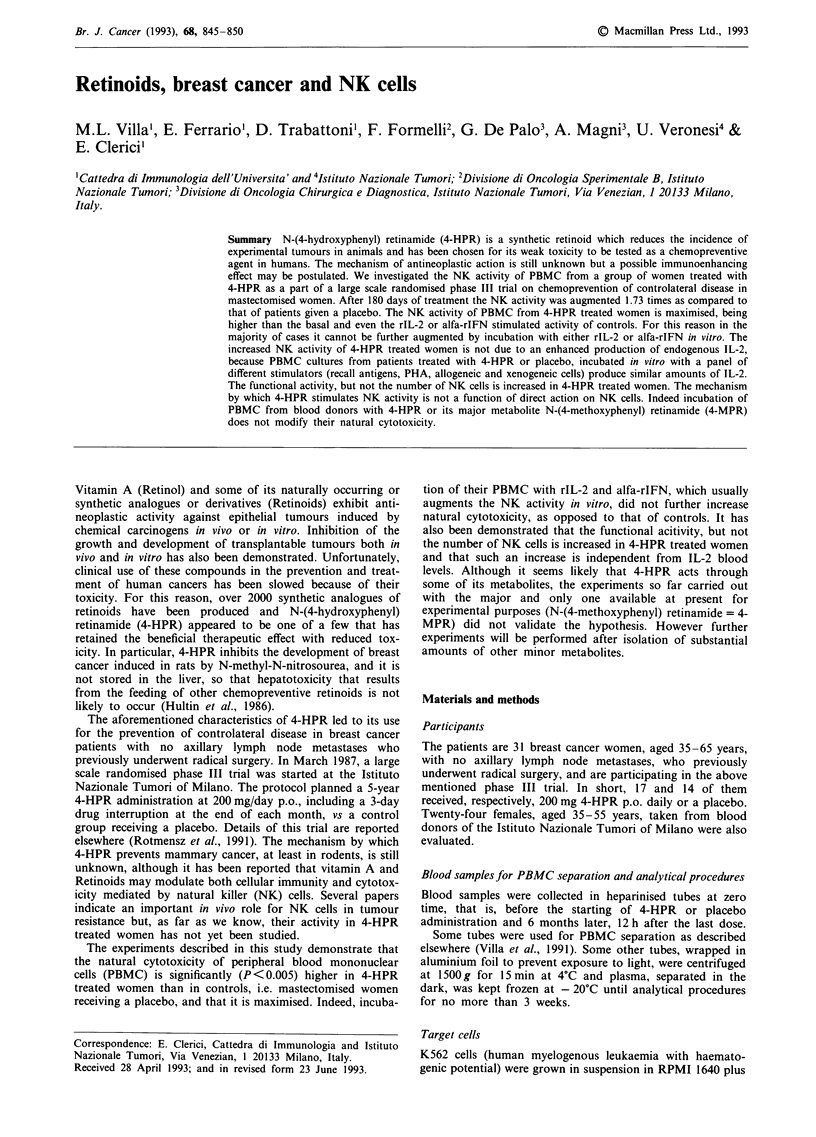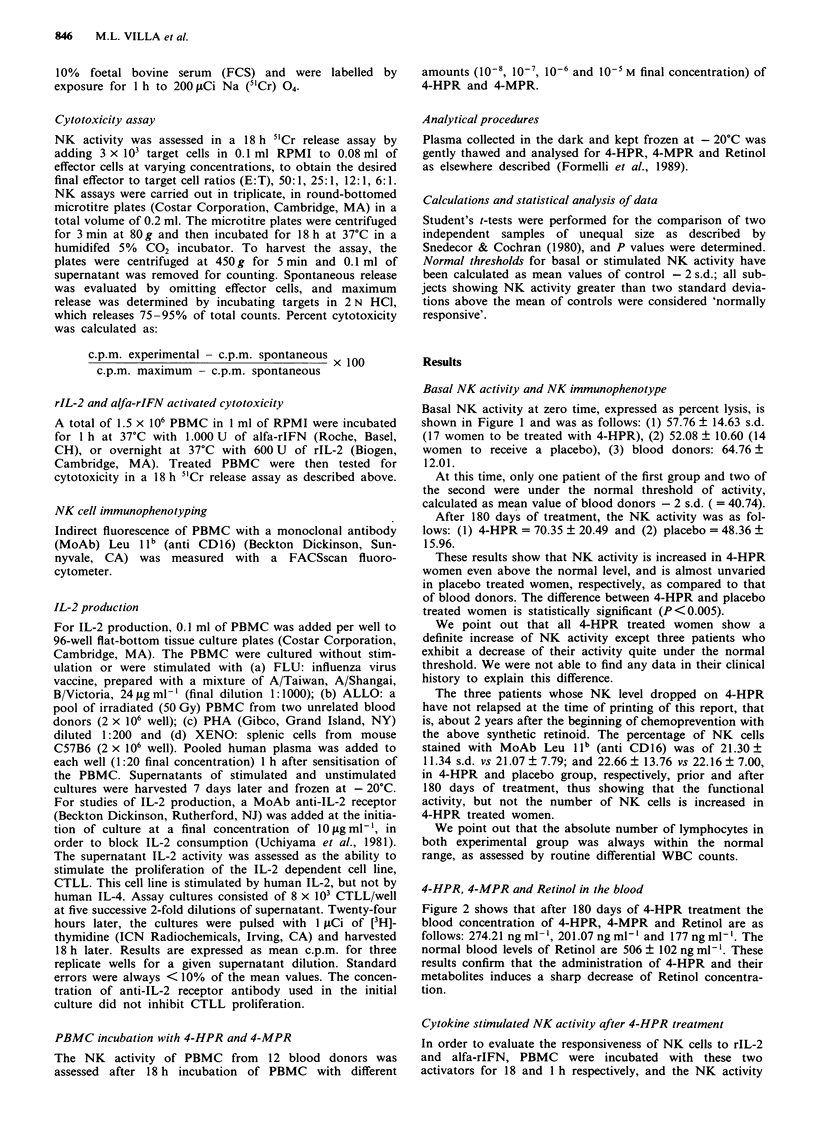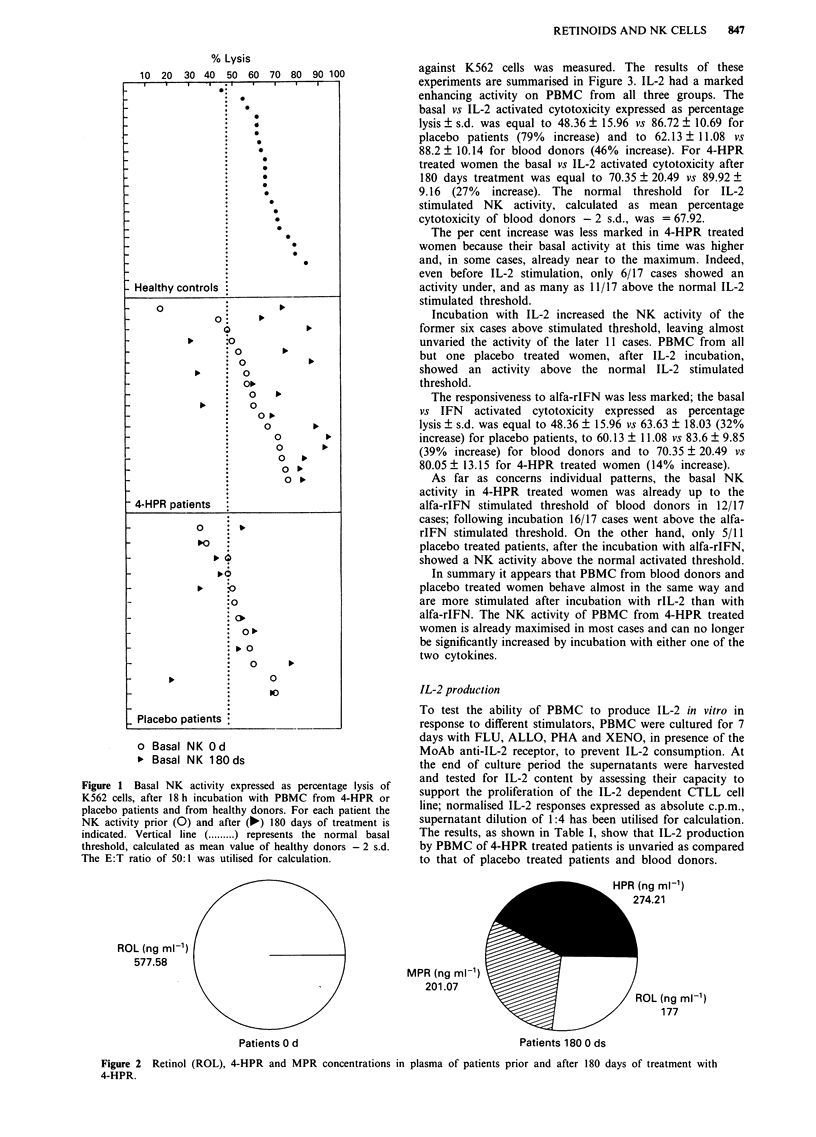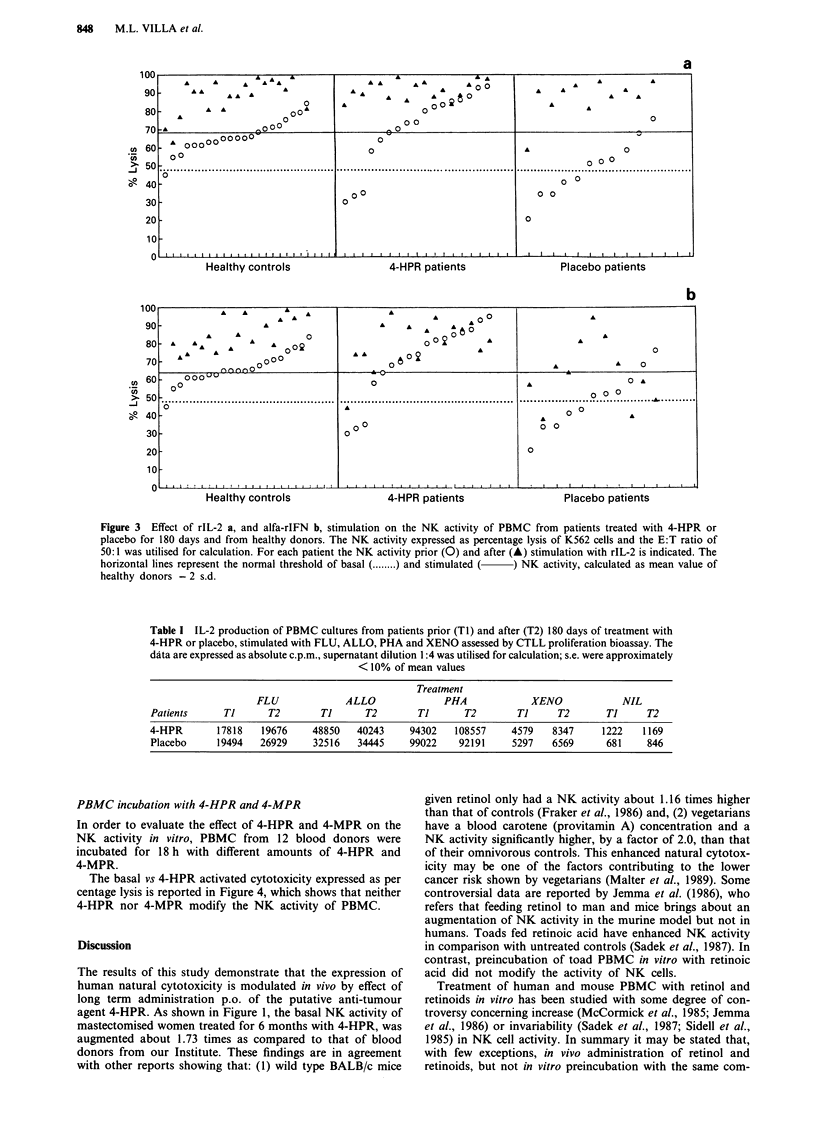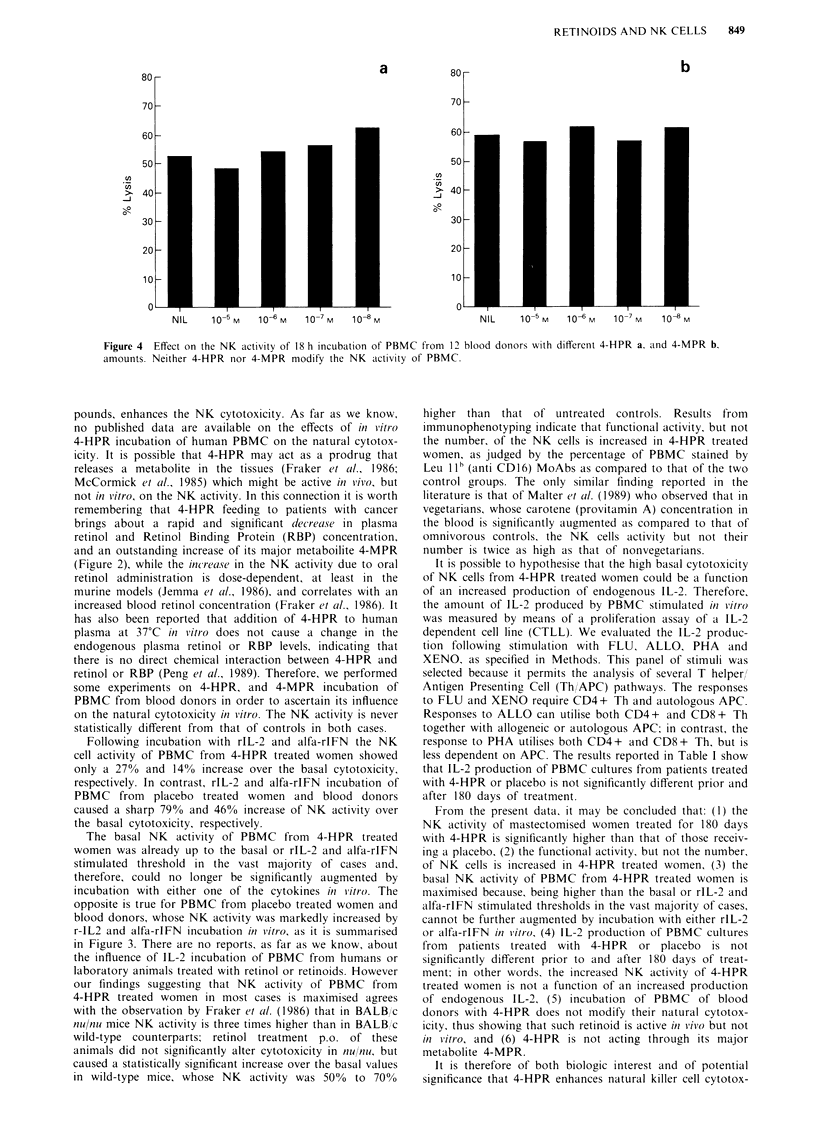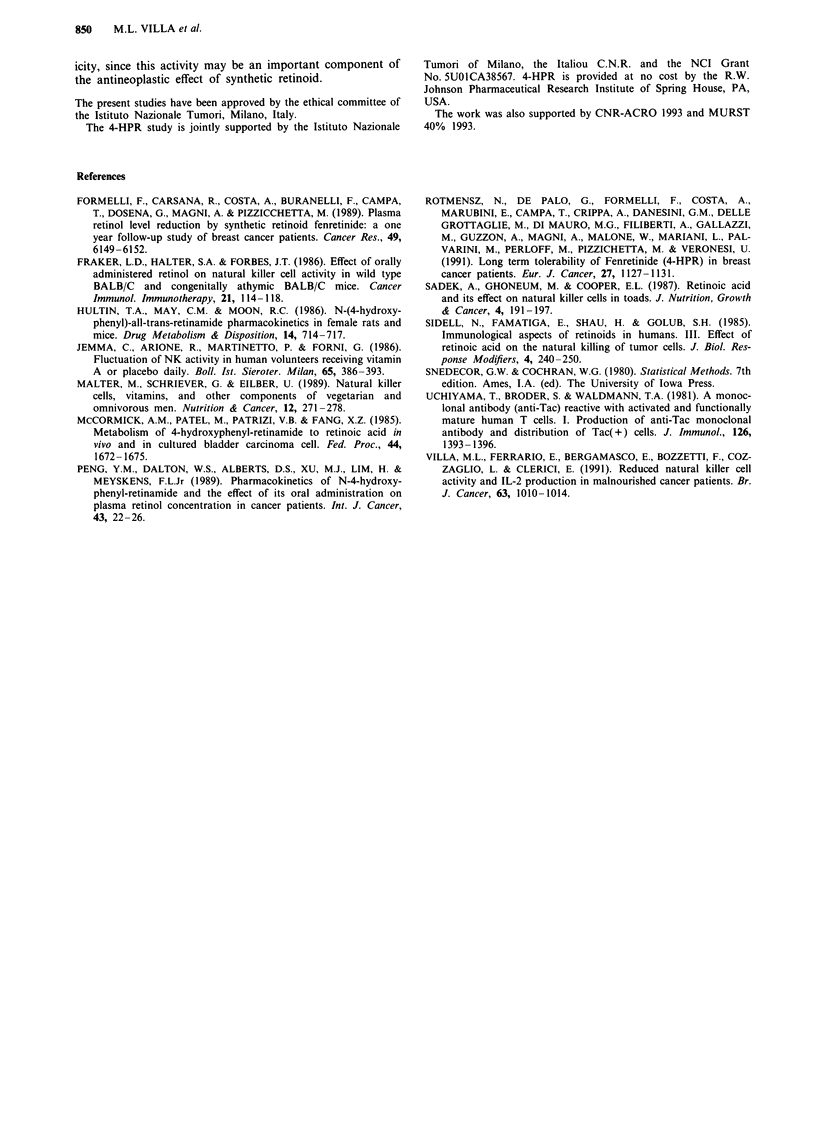# Retinoids, breast cancer and NK cells.

**DOI:** 10.1038/bjc.1993.443

**Published:** 1993-11

**Authors:** M. L. Villa, E. Ferrario, D. Trabattoni, F. Formelli, G. De Palo, A. Magni, U. Veronesi, E. Clerici

**Affiliations:** Cattedra di Immunologia dell'Università, Milano, Italy.

## Abstract

N-(4-hydroxyphenyl) retinamide (4-HPR) is a synthetic retinoid which reduces the incidence of experimental tumours in animals and has been chosen for its weak toxicity to be tested as a chemopreventive agent in humans. The mechanism of antineoplastic action is still unknown but a possible immunoenhancing effect may be postulated. We investigated the NK activity of PBMC from a group of women treated with 4-HPR as a part of a large scale randomised phase III trial on chemoprevention of contralateral disease in mastectomised women. After 180 days of treatment the NK activity was augmented 1.73 times as compared to that of patients given a placebo. The NK activity of PBMC from 4-HPR treated women is maximised, being higher than the basal and even the rIL-2 or alfa-rIFN stimulated activity of controls. For this reason in the majority of cases it cannot be further augmented by incubation with either rIL-2 or alfa-rIFN in vitro. The increased NK activity of 4-HPR treated women is not due to an enhanced production of endogenous IL-2, because PBMC cultures from patients treated with 4-HPR or placebo, incubated in vitro with a panel of different stimulators (recall antigens, PHA, allogeneic and xenogeneic cells) produce similar amounts of IL-2. The functional activity, but not the number of NK cells is increased in 4-HPR treated women. The mechanism by which 4-HPR stimulates NK activity is not a function of direct action on NK cells. Indeed incubation of PBMC from blood donors with 4-HPR or its major metabolite N-(4-methoxyphenyl) retinamide (4-MPR) does not modify their natural cytotoxicity.


Br. J. Cancer (1993), 68, 845–850 © Macmillan Press Ltd., 1993

### Retinoids, breast cancer and NK cells

M.L. Villa[1], E. Ferrario[1], D. Trabattoni[1], F. Formelli[2], G. De Palo[3], A. Magni[3], U. Veronesi[4] & E. Clerici[1]

[1]*Cattedra di Immunologia dell'Universita' and* [4]*Istituto Nazionale Tumori;* [2]*Divisione di Oncologia Sperimentale B, Istituto Nazionale Tumori;* [3]*Divisione di Oncologia Chirurgica e Diagnostica, Istituto Nazionale Tumori, Via Venezian, 1 20133 Milano, Italy.*

**Summary** N-(4-hydroxyphenyl) retinamide (4-HPR) is a synthetic retinoid which reduces the incidence of experimental tumours in animals and has been chosen for its weak toxicity to be tested as a chemopreventive agent in humans. The mechanism of antineoplastic action is still unknown but a possible immunoenhancing effect may be postulated. We investigated the NK activity of PBMC from a group of women treated with 4-HPR as a part of a large scale randomised phase III trial on chemoprevention of controlateral disease in mastectomised women. After 180 days of treatment the NK activity was augmented 1.73 times as compared to that of patients given a placebo. The NK activity of PBMC from 4-HPR treated women is maximised, being higher than the basal and even the rIL-2 or alfa-rIFN stimulated activity of controls. For this reason in the majority of cases it cannot be further augmented by incubation with either rIL-2 or alfa-rIFN *in vitro*. The increased NK activity of 4-HPR treated women is not due to an enhanced production of endogenous IL-2, because PBMC cultures from patients treated with 4-HPR or placebo, incubated *in vitro* with a panel of different stimulators (recall antigens, PHA, allogeneic and xenogeneic cells) produce similar amounts of IL-2. The functional activity, but not the number of NK cells is increased in 4-HPR treated women. The mechanism by which 4-HPR stimulates NK activity is not a function of direct action on NK cells. Indeed incubation of PBMC from blood donors with 4-HPR or its major metabolite N-(4-methoxyphenyl) retinamide (4-MPR) does not modify their natural cytotoxicity.

Vitamin A (Retinol) and some of its naturally occurring or synthetic analogues or derivatives (Retinoids) exhibit antineoplastic activity against epithelial tumours induced by chemical carcinogens *in vivo* or *in vitro*. Inhibition of the growth and development of transplantable tumours both *in vivo* and *in vitro* has also been demonstrated. Unfortunately, clinical use of these compounds in the prevention and treatment of human cancers has been slowed because of their toxicity. For this reason, over 2000 synthetic analogues of retinoids have been produced and N-(4-hydroxyphenyl) retinamide (4-HPR) appeared to be one of a few that has retained the beneficial therapeutic effect with reduced toxicity. In particular, 4-HPR inhibits the development of breast cancer induced in rats by N-methyl-N-nitrosourea, and it is not stored in the liver, so that hepatotoxicity that results from the feeding of other chemopreventive retinoids is not likely to occur (Hultin *et al.*, 1986).

The aforementioned characteristics of 4-HPR led to its use for the prevention of controlateral disease in breast cancer patients with no axillary lymph node metastases who previously underwent radical surgery. In March 1987, a large scale randomised phase III trial was started at the Istituto Nazionale Tumori of Milano. The protocol planned a 5-year 4-HPR administration at 200 mg/day p.o., including a 3-day drug interruption at the end of each month, *vs* a control group receiving a placebo. Details of this trial are reported elsewhere (Rotmensz *et al.*, 1991). The mechanism by which 4-HPR prevents mammary cancer, at least in rodents, is still unknown, although it has been reported that vitamin A and Retinoids may modulate both cellular immunity and cytotoxicity mediated by natural killer (NK) cells. Several papers indicate an important *in vivo* role for NK cells in tumour resistance but, as far as we know, their activity in 4-HPR treated women has not yet been studied.

The experiments described in this study demonstrate that the natural cytotoxicity of peripheral blood mononuclear cells (PBMC) is significantly ($P<0.005$) higher in 4-HPR treated women than in controls, i.e. mastectomised women receiving a placebo, and that it is maximised. Indeed, incubation of their PBMC with rIL-2 and alfa-rIFN, which usually augments the NK activity *in vitro*, did not further increase natural cytotoxicity, as opposed to that of controls. It has also been demonstrated that the functional acitivity, but not the number of NK cells is increased in 4-HPR treated women and that such an increase is independent from IL-2 blood levels. Although it seems likely that 4-HPR acts through some of its metabolites, the experiments so far carried out with the major and only one available at present for experimental purposes (N-(4-methoxyphenyl) retinamide = 4-MPR) did not validate the hypothesis. However further experiments will be performed after isolation of substantial amounts of other minor metabolites.

Correspondence: E. Clerici, Cattedra di Immunologia and Istituto Nazionale Tumori, Via Venezian, 1 20133 Milano, Italy.
Received 28 April 1993; and in revised form 23 June 1993.

### Materials and methods

### *Participants*

The patients are 31 breast cancer women, aged 35–65 years, with no axillary lymph node metastases, who previously underwent radical surgery, and are participating in the above mentioned phase III trial. In short, 17 and 14 of them received, respectively, 200 mg 4-HPR p.o. daily or a placebo. Twenty-four females, aged 35–55 years, taken from blood donors of the Istituto Nazionale Tumori of Milano were also evaluated.

### *Blood samples for PBMC separation and analytical procedures*

Blood samples were collected in heparinised tubes at zero time, that is, before the starting of 4-HPR or placebo administration and 6 months later, 12 h after the last dose.

Some tubes were used for PBMC separation as described elsewhere (Villa *et al.*, 1991). Some other tubes, wrapped in aluminium foil to prevent exposure to light, were centrifuged at 1500 *g* for 15 min at 4°C and plasma, separated in the dark, was kept frozen at − 20°C until analytical procedures for no more than 3 weeks.

### *Target cells*

K562 cells (human myelogenous leukaemia with haematogenic potential) were grown in suspension in RPMI 1640 plus

10% foetal bovine serum (FCS) and were labelled by exposure for 1 h to 200 $\mu$Ci Na ($^{51}$Cr) $O_4$.

*Cytotoxicity assay*

NK activity was assessed in a 18 h $^{51}$Cr release assay by adding $3 \times 10^3$ target cells in 0.1 ml RPMI to 0.08 ml of effector cells at varying concentrations, to obtain the desired final effector to target cell ratios (E:T), 50:1, 25:1, 12:1, 6:1. NK assays were carried out in triplicate, in round-bottomed microtitre plates (Costar Corporation, Cambridge, MA) in a total volume of 0.2 ml. The microtitre plates were centrifuged for 3 min at 80 *g* and then incubated for 18 h at 37°C in a humidifed 5% $CO_2$ incubator. To harvest the assay, the plates were centrifuged at 450 *g* for 5 min and 0.1 ml of supernatant was removed for counting. Spontaneous release was evaluated by omitting effector cells, and maximum release was determined by incubating targets in 2 N HCl, which releases 75–95% of total counts. Percent cytotoxicity was calculated as:

$$\frac{\text{c.p.m. experimental} - \text{c.p.m. spontaneous}}{\text{c.p.m. maximum} - \text{c.p.m. spontaneous}} \times 100$$

*rIL-2 and alfa-rIFN activated cytotoxicity*

A total of $1.5 \times 10^6$ PBMC in 1 ml of RPMI were incubated for 1 h at 37°C with 1.000 U of alfa-rIFN (Roche, Basel, CH), or overnight at 37°C with 600 U of rIL-2 (Biogen, Cambridge, MA). Treated PBMC were then tested for cytotoxicity in a 18 h $^{51}$Cr release assay as described above.

*NK cell immunophenotyping*

Indirect fluorescence of PBMC with a monoclonal antibody (MoAb) Leu $11^b$ (anti CD16) (Beckton Dickinson, Sunnyvale, CA) was measured with a FACSscan fluorocytometer.

*IL-2 production*

For IL-2 production, 0.1 ml of PBMC was added per well to 96-well flat-bottom tissue culture plates (Costar Corporation, Cambridge, MA). The PBMC were cultured without stimulation or were stimulated with (a) FLU: influenza virus vaccine, prepared with a mixture of A/Taiwan, A/Shangai, B/Victoria, 24 $\mu$g ml$^{-1}$ (final dilution 1:1000); (b) ALLO: a pool of irradiated (50 Gy) PBMC from two unrelated blood donors ($2 \times 10^6$ well); (c) PHA (Gibco, Grand Island, NY) diluted 1:200 and (d) XENO: splenic cells from mouse C57B6 ($2 \times 10^6$ well). Pooled human plasma was added to each well (1:20 final concentration) 1 h after sensitisation of the PBMC. Supernatants of stimulated and unstimulated cultures were harvested 7 days later and frozen at − 20°C. For studies of IL-2 production, a MoAb anti-IL-2 receptor (Beckton Dickinson, Rutherford, NJ) was added at the initiation of culture at a final concentration of 10 $\mu$g ml$^{-1}$, in order to block IL-2 consumption (Uchiyama *et al.*, 1981). The supernatant IL-2 activity was assessed as the ability to stimulate the proliferation of the IL-2 dependent cell line, CTLL. This cell line is stimulated by human IL-2, but not by human IL-4. Assay cultures consisted of $8 \times 10^3$ CTLL/well at five successive 2-fold dilutions of supernatant. Twenty-four hours later, the cultures were pulsed with 1 $\mu$Ci of [$^3$H]-thymidine (ICN Radiochemicals, Irving, CA) and harvested 18 h later. Results are expressed as mean c.p.m. for three replicate wells for a given supernatant dilution. Standard errors were always $<10\%$ of the mean values. The concentration of anti-IL-2 receptor antibody used in the initial culture did not inhibit CTLL proliferation.

*PBMC incubation with 4-HPR and 4-MPR*

The NK activity of PBMC from 12 blood donors was assessed after 18 h incubation of PBMC with different amounts ($10^{-8}$, $10^{-7}$, $10^{-6}$ and $10^{-5}$ M final concentration) of 4-HPR and 4-MPR.

*Analytical procedures*

Plasma collected in the dark and kept frozen at − 20°C was gently thawed and analysed for 4-HPR, 4-MPR and Retinol as elsewhere described (Formelli *et al.*, 1989).

*Calculations and statistical analysis of data*

Student's *t*-tests were performed for the comparison of two independent samples of unequal size as described by Snedecor & Cochran (1980), and *P* values were determined. *Normal thresholds* for basal or stimulated NK activity have been calculated as mean values of control − 2 s.d.; all subjects showing NK activity greater than two standard deviations above the mean of controls were considered 'normally responsive'.

### Results

*Basal NK activity and NK immunophenotype*

Basal NK activity at zero time, expressed as percent lysis, is shown in Figure 1 and was as follows: (1) 57.76 ± 14.63 s.d. (17 women to be treated with 4-HPR), (2) 52.08 ± 10.60 (14 women to receive a placebo), (3) blood donors: 64.76 ± 12.01.

At this time, only one patient of the first group and two of the second were under the normal threshold of activity, calculated as mean value of blood donors − 2 s.d. ( = 40.74).

After 180 days of treatment, the NK activity was as follows: (1) 4-HPR = 70.35 ± 20.49 and (2) placebo = 48.36 ± 15.96.

These results show that NK activity is increased in 4-HPR women even above the normal level, and is almost unvaried in placebo treated women, respectively, as compared to that of blood donors. The difference between 4-HPR and placebo treated women is statistically significant ($P<0.005$).

We point out that all 4-HPR treated women show a definite increase of NK activity except three patients who exhibit a decrease of their activity quite under the normal threshold. We were not able to find any data in their clinical history to explain this difference.

The three patients whose NK level dropped on 4-HPR have not relapsed at the time of printing of this report, that is, about 2 years after the beginning of chemoprevention with the above synthetic retinoid. The percentage of NK cells stained with MoAb Leu $11^b$ (anti CD16) was of 21.30 ± 11.34 s.d. *vs* 21.07 ± 7.79; and 22.66 ± 13.76 *vs* 22.16 ± 7.00, in 4-HPR and placebo group, respectively, prior and after 180 days of treatment, thus showing that the functional activity, but not the number of NK cells is increased in 4-HPR treated women.

We point out that the absolute number of lymphocytes in both experimental group was always within the normal range, as assessed by routine differential WBC counts.

*4-HPR, 4-MPR and Retinol in the blood*

Figure 2 shows that after 180 days of 4-HPR treatment the blood concentration of 4-HPR, 4-MPR and Retinol are as follows: 274.21 ng ml$^{-1}$, 201.07 ng ml$^{-1}$ and 177 ng ml$^{-1}$. The normal blood levels of Retinol are 506 ± 102 ng ml$^{-1}$. These results confirm that the administration of 4-HPR and their metabolites induces a sharp decrease of Retinol concentration.

*Cytokine stimulated NK activity after 4-HPR treatment*

In order to evaluate the responsiveness of NK cells to rIL-2 and alfa-rIFN, PBMC were incubated with these two activators for 18 and 1 h respectively, and the NK activity

**Figure 1** Basal NK activity expressed as percentage lysis of K562 cells, after 18 h incubation with PBMC from 4-HPR or placebo patients and from healthy donors. For each patient the NK activity prior (O) and after (▶) 180 days of treatment is indicated. Vertical line (.........) represents the normal basal threshold, calculated as mean value of healthy donors − 2 s.d. The E:T ratio of 50:1 was utilised for calculation.

against K562 cells was measured. The results of these experiments are summarised in Figure 3. IL-2 had a marked enhancing activity on PBMC from all three groups. The basal *vs* IL-2 activated cytotoxicity expressed as percentage lysis ± s.d. was equal to 48.36 ± 15.96 *vs* 86.72 ± 10.69 for placebo patients (79% increase) and to 62.13 ± 11.08 *vs* 88.2 ± 10.14 for blood donors (46% increase). For 4-HPR treated women the basal *vs* IL-2 activated cytotoxicity after 180 days treatment was equal to 70.35 ± 20.49 *vs* 89.92 ± 9.16 (27% increase). The normal threshold for IL-2 stimulated NK activity, calculated as mean percentage cytotoxicity of blood donors − 2 s.d., was = 67.92.

The per cent increase was less marked in 4-HPR treated women because their basal activity at this time was higher and, in some cases, already near to the maximum. Indeed, even before IL-2 stimulation, only 6/17 cases showed an activity under, and as many as 11/17 above the normal IL-2 stimulated threshold.

Incubation with IL-2 increased the NK activity of the former six cases above stimulated threshold, leaving almost unvaried the activity of the later 11 cases. PBMC from all but one placebo treated women, after IL-2 incubation, showed an activity above the normal IL-2 stimulated threshold.

The responsiveness to alfa-rIFN was less marked; the basal *vs* IFN activated cytotoxicity expressed as percentage lysis ± s.d. was equal to 48.36 ± 15.96 *vs* 63.63 ± 18.03 (32% increase) for placebo patients, to 60.13 ± 11.08 *vs* 83.6 ± 9.85 (39% increase) for blood donors and to 70.35 ± 20.49 *vs* 80.05 ± 13.15 for 4-HPR treated women (14% increase).

As far as concerns individual patterns, the basal NK activity in 4-HPR treated women was already up to the alfa-rIFN stimulated threshold of blood donors in 12/17 cases; following incubation 16/17 cases went above the alfa-rIFN stimulated threshold. On the other hand, only 5/11 placebo treated patients, after the incubation with alfa-rIFN, showed a NK activity above the normal activated threshold.

In summary it appears that PBMC from blood donors and placebo treated women behave almost in the same way and are more stimulated after incubation with rIL-2 than with alfa-rIFN. The NK activity of PBMC from 4-HPR treated women is already maximised in most cases and can no longer be significantly increased by incubation with either one of the two cytokines.

*IL-2 production*

To test the ability of PBMC to produce IL-2 *in vitro* in response to different stimulators, PBMC were cultured for 7 days with FLU, ALLO, PHA and XENO, in presence of the MoAb anti-IL-2 receptor, to prevent IL-2 consumption. At the end of culture period the supernatants were harvested and tested for IL-2 content by assessing their capacity to support the proliferation of the IL-2 dependent CTLL cell line; normalised IL-2 responses expressed as absolute c.p.m., supernatant dilution of 1:4 has been utilised for calculation. The results, as shown in Table I, show that IL-2 production by PBMC of 4-HPR treated patients is unvaried as compared to that of placebo treated patients and blood donors.

**Figure 2** Retinol (ROL), 4-HPR and MPR concentrations in plasma of patients prior and after 180 days of treatment with 4-HPR.

**Figure 3** Effect of rIL-2 **a**, and alfa-rIFN **b**, stimulation on the NK activity of PBMC from patients treated with 4-HPR or placebo for 180 days and from healthy donors. The NK activity expressed as percentage lysis of K562 cells and the E:T ratio of 50:1 was utilised for calculation. For each patient the NK activity prior (O) and after (▲) stimulation with rIL-2 is indicated. The horizontal lines represent the normal threshold of basal (........) and stimulated (———) NK activity, calculated as mean value of healthy donors − 2 s.d.

**Table I** IL-2 production of PBMC cultures from patients prior (T1) and after (T2) 180 days of treatment with 4-HPR or placebo, stimulated with FLU, ALLO, PHA and XENO assessed by CTLL proliferation bioassay. The data are expressed as absolute c.p.m., supernatant dilution 1:4 was utilised for calculation; s.e. were approximately <10% of mean values

| | *Treatment* | | | | | | | | | |
|---|---|---|---|---|---|---|---|---|---|---|
| | *FLU* | | *ALLO* | | *PHA* | | *XENO* | | *NIL* | |
| *Patients* | *T1* | *T2* | *T1* | *T2* | *T1* | *T2* | *T1* | *T2* | *T1* | *T2* |
| 4-HPR | 17818 | 19676 | 48850 | 40243 | 94302 | 108557 | 4579 | 8347 | 1222 | 1169 |
| Placebo | 19494 | 26929 | 32516 | 34445 | 99022 | 92191 | 5297 | 6569 | 681 | 846 |

*PBMC incubation with 4-HPR and 4-MPR*

In order to evaluate the effect of 4-HPR and 4-MPR on the NK activity *in vitro*, PBMC from 12 blood donors were incubated for 18 h with different amounts of 4-HPR and 4-MPR.

The basal *vs* 4-HPR activated cytotoxicity expressed as per centage lysis is reported in Figure 4, which shows that neither 4-HPR nor 4-MPR modify the NK activity of PBMC.

### Discussion

The results of this study demonstrate that the expression of human natural cytotoxicity is modulated *in vivo* by effect of long term administration p.o. of the putative anti-tumour agent 4-HPR. As shown in Figure 1, the basal NK activity of mastectomised women treated for 6 months with 4-HPR, was augmented about 1.73 times as compared to that of blood donors from our Institute. These findings are in agreement with other reports showing that: (1) wild type BALB/c mice given retinol only had a NK activity about 1.16 times higher than that of controls (Fraker *et al.*, 1986) and, (2) vegetarians have a blood carotene (provitamin A) concentration and a NK activity significantly higher, by a factor of 2.0, than that of their omnivorous controls. This enhanced natural cytotoxicity may be one of the factors contributing to the lower cancer risk shown by vegetarians (Malter *et al.*, 1989). Some controversial data are reported by Jemma *et al.* (1986), who refers that feeding retinol to man and mice brings about an augmentation of NK activity in the murine model but not in humans. Toads fed retinoic acid have enhanced NK activity in comparison with untreated controls (Sadek *et al.*, 1987). In contrast, preincubation of toad PBMC *in vitro* with retinoic acid did not modify the activity of NK cells.

Treatment of human and mouse PBMC with retinol and retinoids *in vitro* has been studied with some degree of controversy concerning increase (McCormick *et al.*, 1985; Jemma *et al.*, 1986) or invariability (Sadek *et al.*, 1987; Sidell *et al.*, 1985) in NK cell activity. In summary it may be stated that, with few exceptions, *in vivo* administration of retinol and retinoids, but not *in vitro* preincubation with the same com-

**Figure 4** Effect on the NK activity of 18 h incubation of PBMC from 12 blood donors with different 4-HPR **a**, and 4-MPR **b**, amounts. Neither 4-HPR nor 4-MPR modify the NK activity of PBMC.

pounds, enhances the NK cytotoxicity. As far as we know, no published data are available on the effects of *in vitro* 4-HPR incubation of human PBMC on the natural cytotoxicity. It is possible that 4-HPR may act as a prodrug that releases a metabolite in the tissues (Fraker *et al.*, 1986; McCormick *et al.*, 1985) which might be active *in vivo*, but not *in vitro*, on the NK activity. In this connection it is worth remembering that 4-HPR feeding to patients with cancer brings about a rapid and significant *decrease* in plasma retinol and Retinol Binding Protein (RBP) concentration, and an outstanding increase of its major metaboilite 4-MPR (Figure 2), while the *increase* in the NK activity due to oral retinol administration is dose-dependent, at least in the murine models (Jemma *et al.*, 1986), and correlates with an increased blood retinol concentration (Fraker *et al.*, 1986). It has also been reported that addition of 4-HPR to human plasma at 37°C *in vitro* does not cause a change in the endogenous plasma retinol or RBP levels, indicating that there is no direct chemical interaction between 4-HPR and retinol or RBP (Peng *et al.*, 1989). Therefore, we performed some experiments on 4-HPR, and 4-MPR incubation of PBMC from blood donors in order to ascertain its influence on the natural cytotoxicity *in vitro*. The NK activity is never statistically different from that of controls in both cases.

Following incubation with rIL-2 and alfa-rIFN the NK cell activity of PBMC from 4-HPR treated women showed only a 27% and 14% increase over the basal cytotoxicity, respectively. In contrast, rIL-2 and alfa-rIFN incubation of PBMC from placebo treated women and blood donors caused a sharp 79% and 46% increase of NK activity over the basal cytotoxicity, respectively.

The basal NK activity of PBMC from 4-HPR treated women was already up to the basal or rIL-2 and alfa-rIFN stimulated threshold in the vast majority of cases and, therefore, could no longer be significantly augmented by incubation with either one of the cytokines *in vitro*. The opposite is true for PBMC from placebo treated women and blood donors, whose NK activity was markedly increased by r-IL2 and alfa-rIFN incubation *in vitro*, as it is summarised in Figure 3. There are no reports, as far as we know, about the influence of IL-2 incubation of PBMC from humans or laboratory animals treated with retinol or retinoids. However our findings suggesting that NK activity of PBMC from 4-HPR treated women in most cases is maximised agrees with the observation by Fraker *et al.* (1986) that in BALB/c *nu/nu* mice NK activity is three times higher than in BALB/c wild-type counterparts; retinol treatment p.o. of these animals did not significantly alter cytotoxicity in *nu/nu*, but caused a statistically significant increase over the basal values in wild-type mice, whose NK activity was 50% to 70% higher than that of untreated controls. Results from immunophenotyping indicate that functional activity, but not the number, of the NK cells is increased in 4-HPR treated women, as judged by the percentage of PBMC stained by Leu $11^b$ (anti CD16) MoAbs as compared to that of the two control groups. The only similar finding reported in the literature is that of Malter *et al.* (1989) who observed that in vegetarians, whose carotene (provitamin A) concentration in the blood is significantly augmented as compared to that of omnivorous controls, the NK cells activity but not their number is twice as high as that of nonvegetarians.

It is possible to hypothesise that the high basal cytotoxicity of NK cells from 4-HPR treated women could be a function of an increased production of endogenous IL-2. Therefore, the amount of IL-2 produced by PBMC stimulated *in vitro* was measured by means of a proliferation assay of a IL-2 dependent cell line (CTLL). We evaluated the IL-2 production following stimulation with FLU, ALLO, PHA and XENO, as specified in Methods. This panel of stimuli was selected because it permits the analysis of several T helper/Antigen Presenting Cell (Th/APC) pathways. The responses to FLU and XENO require CD4+ Th and autologous APC. Responses to ALLO can utilise both CD4+ and CD8+ Th together with allogeneic or autologous APC; in contrast, the response to PHA utilises both CD4+ and CD8+ Th, but is less dependent on APC. The results reported in Table I show that IL-2 production of PBMC cultures from patients treated with 4-HPR or placebo is not significantly different prior and after 180 days of treatment.

From the present data, it may be concluded that: (1) the NK activity of mastectomised women treated for 180 days with 4-HPR is significantly higher than that of those receiving a placebo, (2) the functional activity, but not the number, of NK cells is increased in 4-HPR treated women, (3) the basal NK activity of PBMC from 4-HPR treated women is maximised because, being higher than the basal or rIL-2 and alfa-rIFN stimulated thresholds in the vast majority of cases, cannot be further augmented by incubation with either rIL-2 or alfa-rIFN *in vitro*, (4) IL-2 production of PBMC cultures from patients treated with 4-HPR or placebo is not significantly different prior to and after 180 days of treatment; in other words, the increased NK activity of 4-HPR treated women is not a function of an increased production of endogenous IL-2, (5) incubation of PBMC of blood donors with 4-HPR does not modify their natural cytotoxicity, thus showing that such retinoid is active *in vivo* but not *in vitro*, and (6) 4-HPR is not acting through its major metabolite 4-MPR.

It is therefore of both biologic interest and of potential significance that 4-HPR enhances natural killer cell cytotoxicity, since this activity may be an important component of the antineoplastic effect of synthetic retinoid.

The present studies have been approved by the ethical committee of the Istituto Nazionale Tumori, Milano, Italy.

The 4-HPR study is jointly supported by the Istituto Nazionale Tumori of Milano, the Italiou C.N.R. and the NCI Grant No. 5U01CA38567. 4-HPR is provided at no cost by the R.W. Johnson Pharmaceutical Research Institute of Spring House, PA, USA.

The work was also supported by CNR-ACRO 1993 and MURST 40% 1993.

### References

FORMELLI, F., CARSANA, R., COSTA, A., BURANELLI, F., CAMPA, T., DOSENA, G., MAGNI, A. & PIZZICCHETTA, M. (1989). Plasma retinol level reduction by synthetic retinoid fenretinide: a one year follow-up study of breast cancer patients. *Cancer Res.*, **49**, 6149-6152.

FRAKER, L.D., HALTER, S.A. & FORBES, J.T. (1986). Effect of orally administered retinol on natural killer cell activity in wild type BALB/C and congenitally athymic BALB/C mice. *Cancer Immunol. Immunotherapy*, **21**, 114-118.

HULTIN, T.A., MAY, C.M. & MOON, R.C. (1986). N-(4-hydroxyphenyl)-all-trans-retinamide pharmacokinetics in female rats and mice. *Drug Metabolism & Disposition*, **14**, 714-717.

JEMMA, C., ARIONE, R., MARTINETTO, P. & FORNI, G. (1986). Fluctuation of NK activity in human volunteers receiving vitamin A or placebo daily. *Boll. Ist. Sieroter. Milan*, **65**, 386-393.

MALTER, M., SCHRIEVER, G. & EILBER, U. (1989). Natural killer cells, vitamins, and other components of vegetarian and omnivorous men. *Nutrition & Cancer*, **12**, 271-278.

MCCORMICK, A.M., PATEL, M., PATRIZI, V.B. & FANG, X.Z. (1985). Metabolism of 4-hydroxyphenyl-retinamide to retinoic acid *in vivo* and in cultured bladder carcinoma cell. *Fed. Proc.*, **44**, 1672-1675.

PENG, Y.M., DALTON, W.S., ALBERTS, D.S., XU, M.J., LIM, H. & MEYSKENS, F.L.Jr (1989). Pharmacokinetics of N-4-hydroxyphenyl-retinamide and the effect of its oral administration on plasma retinol concentration in cancer patients. *Int. J. Cancer*, **43**, 22-26.

ROTMENSZ, N., DE PALO, G., FORMELLI, F., COSTA, A., MARUBINI, E., CAMPA, T., CRIPPA, A., DANESINI, G.M., DELLE GROTTAGLIE, M., DI MAURO, M.G., FILIBERTI, A., GALLAZZI, M., GUZZON, A., MAGNI, A., MALONE, W., MARIANI, L., PALVARINI, M., PERLOFF, M., PIZZICHETTA, M. & VERONESI, U. (1991). Long term tolerability of Fenretinide (4-HPR) in breast cancer patients. *Eur. J. Cancer*, **27**, 1127-1131.

SADEK, A., GHONEUM, M. & COOPER, E.L. (1987). Retinoic acid and its effect on natural killer cells in toads. *J. Nutrition, Growth & Cancer*, **4**, 191-197.

SIDELL, N., FAMATIGA, E., SHAU, H. & GOLUB, S.H. (1985). Immunological aspects of retinoids in humans. III. Effect of retinoic acid on the natural killing of tumor cells. *J. Biol. Response Modifiers*, **4**, 240-250.

SNEDECOR, G.W. & COCHRAN, W.G. (1980). *Statistical Methods*. 7th edition. Ames, I.A. (ed). The University of Iowa Press.

UCHIYAMA, T., BRODER, S. & WALDMANN, T.A. (1981). A monoclonal antibody (anti-Tac) reactive with activated and functionally mature human T cells. I. Production of anti-Tac monoclonal antibody and distribution of Tac(+) cells. *J. Immunol.*, **126**, 1393-1396.

VILLA, M.L., FERRARIO, E., BERGAMASCO, E., BOZZETTI, F., COZZAGLIO, L. & CLERICI, E. (1991). Reduced natural killer cell activity and IL-2 production in malnourished cancer patients. *Br. J. Cancer*, **63**, 1010-1014.